# The Bedside Dietary

**Published:** 1894-06

**Authors:** 


THE BEDSIDE DIETARY. Arranged by Gideon C. Segur, M. D. The Plympton Manufacturing Co., Hartford, Conn.

This is a little pamphlet of six pages, easily carried in the inside coat pocket, containing a series of the most simple receipts for diluted milk, junket, whey, egg and milk junket, clam broth, oyster broth, etc. It is printed only on right-hand pages, the left hand being blank for reception of notes.

